# METTL3-mediated deficiency of lncRNA HAR1A drives non-small cell lung cancer growth and metastasis by promoting ANXA2 stabilization

**DOI:** 10.1038/s41420-024-01965-w

**Published:** 2024-04-30

**Authors:** Xiaodong Ling, Cuicui Qi, Kui Cao, Mengdi Lu, Yingnan Yang, Jinfeng Zhang, Luquan Zhang, Jinhong Zhu, Jianqun Ma

**Affiliations:** 1 Department of Thoracic Surgery, Harbin Medical University Cancer Hospital, 150 Haping Road, Harbin, 150040 Heilongjiang China; 2 Department of Clinical Laboratory, Harbin Medical University Cancer Hospital, 150 Haping Road, Harbin, 150040 Heilongjiang China; 3 Biobank, Harbin Medical University Cancer Hospital, 150 Haping Road, Harbin, 150040 Heilongjiang China

**Keywords:** Non-small-cell lung cancer, Long non-coding RNAs

## Abstract

We previously reported lncRNA *HAR1A* as a tumor suppressor in non-small cell lung cancer (NSCLC). However, the delicate working mechanisms of this lncRNA remain obscure. Herein, we demonstrated that the ectopic expression of *HAR1A* inhibited the proliferation, epithelial-mesenchymal transition (EMT), migration, and invasion of NSCLC cells and enhanced paclitaxel (PTX) sensitivity in vitro and in vivo. We identified the oncogenic protein annexin 2 (ANXA2) as a potential interacting patterner of *HAR1A*. *HAR1A* overexpression enhanced ANXA2 ubiquitination and accelerated its degradation via the ubiquitin–proteasome pathway. We further uncovered that *HAR1A* promoted the interaction between E3 ubiquitin ligase TRIM65 and ANXA2. Moreover, the ANXA2 plasmid transfection could reverse *HAR1A* overexpression-induced decreases in proliferation, migration, and invasion of NSCLC cells and the activity of the NF-κB signaling pathway. Finally, we found that *HAR1A* loss in NSCLC might be attributed to the upregulated METTL3. The m^6^A modification levels of *HAR1A* were increased in cancer cells, while YTHDF2 was responsible for recognizing m^6^A modification in the *HAR1A*, leading to the disintegration of this lncRNA. In conclusion, we found that METTL3-mediated m^6^A modification decreased *HAR1A* in NSCLC. *HAR1A* deficiency, in turn, stimulated tumor growth and metastasis by activating the ANXA2/p65 axis.

## Introduction

Long non-coding RNAs (lncRNAs) are referred to as transcribed RNA molecules consisting of more than 200 nucleotides and lacking protein-coding potential. Although used to be thought of as transcriptional noise or artefactual transcripts, lncRNAs are critical for gene expression by extensively participating in and controlling transcription, splicing, post-translational modification, stability, and translation [1]. Accumulating evidence has highlighted systematical alterations in RNA processing during carcinogenesis, leading to aberrant expression levels of various RNAs such as mRNA, miRNA, tRNA, circRNA, and lncRNAs [2]. Dysregulated lncRNAs can act as oncogenes or tumor suppressors to contribute to tumorigenesis, metastasis, drug resistance, and progression [2]. Elevated LncRNA LINC00942 conferred gastric cancer (GC) chemoresistance by activating the MSI2/c-Myc axis to induce stemness and suppress apoptosis of GC cells [3]. Oncogenic lncRNA SNHG20 was increased in NSCLC tissues and associated with prognosis, which epigenetically inhibited p21 expression to promote tumor cell growth [4]. Tumor-suppressive lncRNA *DRAIC* was reduced in castration-resistant advanced prostate cancer (PC), which inactivated nuclear factor-κB (NF-κB) to inhibit tumor progression [5]. In recent years, lncRNAs have shown potential as diagnostic markers and therapy monitoring in cancer [6, 7]. Moreover, ncRNAs are potential therapeutic targets for cancer due to their complex spatial structures and tumor-promoting and -suppressing functions [8]. Therefore, it is imperative to identify and characterize the regulation and functional significance of novel lncRNAs to understand the fundamental biological processes of tumorigenesis and advance cancer treatments.

lncRNA *Highly Accelerated Region 1A* (*HAR1A*) is a large intergenic non-coding RNA. Our pan-cancer analysis revealed that the expression of *HAR1A* was downregulated in a broad spectrum of cancers, including lung adenocarcinoma (LUAD), lung squamous carcinoma (LUSC), bladder urothelial carcinoma (BLCA), breast invasive carcinoma (BRCA), uterine corpus endometrial carcinoma (CESC), liver hepatocellular carcinoma (LIHC), prostate adenocarcinoma (PRAD), and stomach adenocarcinoma (STAD) [9]. In vitro and in vivo studies demonstrated that *HAR1A* suppressed NSCLC growth [9]. Moreover, lncRNA *HAR1A* was found to retard the progression of oral cancer by inhibiting the ALPK1/BRD7/myosin IIA axis [10]. However, The molecular mechanisms underlying the *HAR1A*’s tumor-suppressing functions are largely unclear. This study aimed to interrogate further the biological functions of *HAR1A* and the molecular mechanisms behind it.

Annexin A2 (ANXA2) is a member of the annexin family, comprising calcium-dependent phospholipid-binding proteins. ANXA2 is overexpressed in several types of cancer, such as hepatocellular carcinoma (HCC) [11], breast cancer [12], and NSCLC [13], and contributes to cancer development and progression. For instance, ANXA2 was activated via sequential phosphorylation by S6K1 and K63-linked polyubiquitination by FBXW10 in HCC tissues from male patients [14]. Activated ANXA2 transferred to the cell membrane to activate the KRAS/MEK/ERK pathway, promoting HCC proliferation and lung metastasis [14]. These findings suggest that ANXA2 may hold promise to be a potential biomarker and therapeutic target for cancer. Moreover, the regulation of ANXA2 on many signaling pathways has been documented, including the β-catenin [15], ERK [14, 16], and NF-κB [17, 18].

N6-methyladenosine (m^6^A) RNA modification is an epigenetic regulatory mechanism for coding and ncRNAs, which plays essential roles in almost all vital biological and pathological processes, including malignant transformations. m^6^A modifications are catalyzed by a m^6^A methyltransferase complex (MTC), comprising the methyltransferase-like 3 (METTL3), METTL14, WTAP, and other regulatory components. Elevated METTL3 has been seen in NSCLC and is involved in cancer progress [19–21]. Therefore, we also explore whether *HAR1A* is subjected to METTL3-dependent m^6^A modification.

Here, we demonstrated that *HAR1A* was downregulated by METTL3-mediated m^6^A modification. *HAR1A*, in part, controlled proliferation, migration, invasion, and EMT of NSCLC cells by boosting the interaction between ANXA2 and its E3 ligase TRIM65 and thereby disintegrating oncogenic ANXA2 to inactivate the NF-κB signaling pathway. Thereby, we identified a novel fine-tuning mechanism interpreting how the loss of *HAR1A* promoted cancer development and how *HAR1A* was decreased in NSCLC.

## Results

### lncRNA *HAR1A* increases NSCLC cells’ response to paclitaxel

We first determined the endogenous expression levels of *HAR1A* in the indicated cell lines. Compared to normal epithelial cells, several cancer cell lines exhibited decreased expression of *HAR1A* (Supplementary Fig. 1a). A549 and H1299 cells were infected with lentiviral vectors expressing *HAR1A* to establish stable cell lines (Supplementary Fig. 1b). The proliferation of A549 and H1299 cells was significantly inhibited by *HAR1A* overexpression (Supplementary Fig. 1c).

**Fig. 1:**
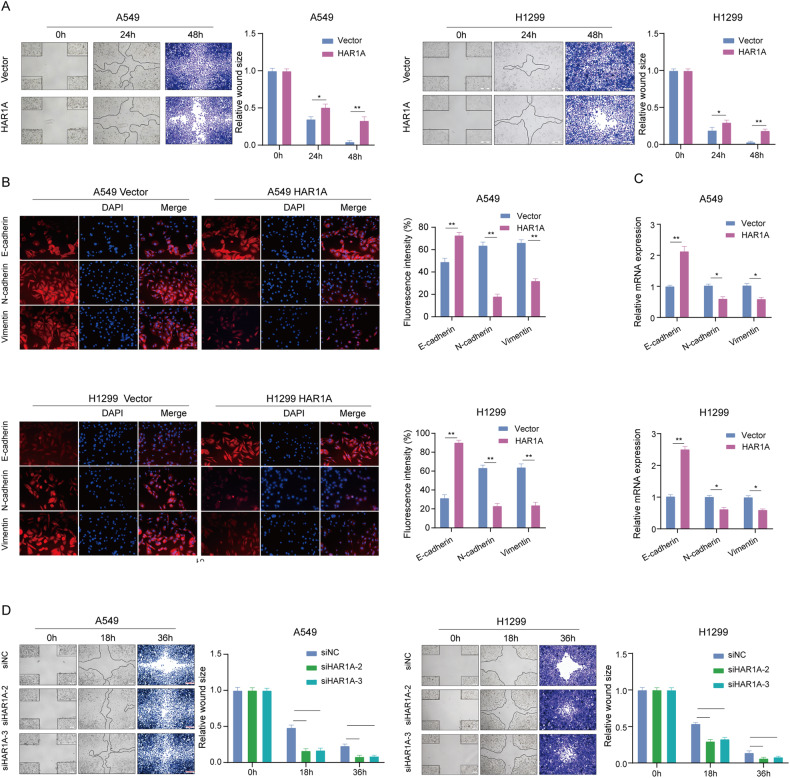
The influences of *HAR1A* on malignant behaviors of NSCLC cells. **A** Representative images and quantifications indicating scratching assay of *HAR1A*-overexpressing A549 and H1299 stable cells and respective empty vector-expressing cells. **B** Immunofluorescent staining was performed to detect the expression of epithelial-mesenchymal transition (EMT) biomarkers, including E-cadherin, N-cadherin, and vimentin. Fluorescence intensity (100%) was measured to represent the expression levels of EMT biomarkers. **C** The mRNA levels of these EMT biomarkers were also evaluated using RT-qPCR. **D** The wound healing assay indicated that gaps almost disappeared in si*HAR1A* cells compared with unfilled gaps in siNC cells at 36h after scratching, suggesting that silencing *HAR1A* promoted NSCLC cell migration. *p < 0.05; **p < 0.01; ***p < 0.001; ****p < 0.0001.

**Fig. 2:**
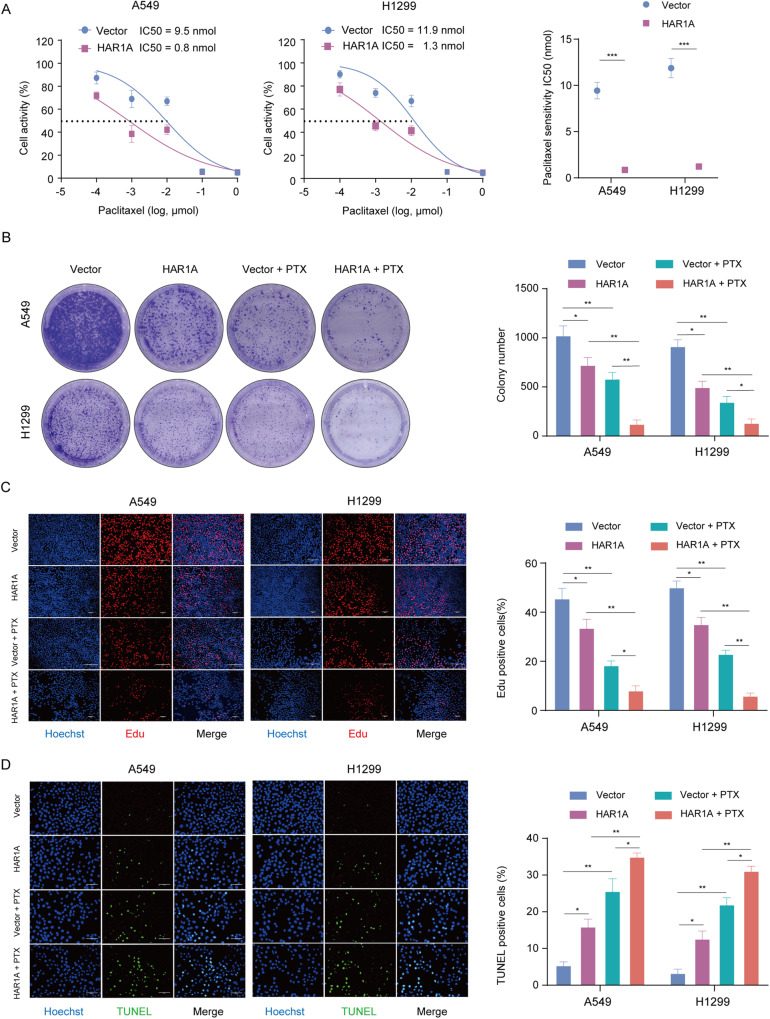
*HAR1A* sensitizes NSCLC cells to paclitaxel (PTX). **A** A549 and H1299 cells were infected lentivirus (LV) empty vectors and LV overexpressing HAR1A (LV-HAR1A) and incubated with increasing concentrations of PTX. Cell viability was checked after 24 h using CCK8 assay. IC50 was calculated in both A549 and H1299 cells. The following experiments were performed using LV-vector and LV-*HAR1A* cells in the presence or absence of PTX. **B** *HAR1A* further aggravated PTX’s colony formation-inhibiting effects on NSCLC cells, suggesting that *HAR1A* might have acted as a chemosensitizer. **C** Edu incorporation assays were used to monitor dividing cells. With the percentage of EdU-labeling cells used as an estimate for proliferation rate, PTX exhibited significantly stronger inhibitory effects on LV-*HAR1A* NSCLC cells than on vector control cells. **D** TUNEL assay revealed that PTX treatment triggered significantly more cells to undergo apoptosis in LV-*HAR1A* NSCLC cells than in vector control cells. *p < 0.05; **p < 0.01; ***p < 0.001; ****p < 0.0001.

Next, the wound healing assay indicated that elevated expression of *HAR1A* also decelerated the migration of NSCLC cells. In contrast to the control group, the created gaps remained unfilled post 48 h (Fig. 1A). Consistently, immunocytostaining and RT-qPCR results demonstrated that cells overexpressing *HAR1A* showed an increase in E-cadherin and decreases in N-cadherin and vimentin, suggesting a reversal of epithelial-mesenchymal transition (EMT) related to reduced migration ability of cells (Fig. 1B, C). We also used siRNA to knock down *HAR1A* in the A549 and H1299 cells (Supplementary Fig. 1d). Silencing *HAR1A* exhibited opposite effects on cancer cells. The downregulation of *HAR1A* significantly prompted the growth and migration of NSCLC cells (Supplementary Fig. 1e and Fig. 1D). Moreover, enforced expression of the lncRNA sensitized NSCLC cells to paclitaxel (PTX) as indicated by IC50 (Fig. 2A). PTX treatment hindered proliferation and induced apoptosis of NSCLC cells. The enforced expression of *HAR1A* enhanced the cytotoxicity of PTX on NSCLC cells as revealed by colony formation, Edu, and TUNEL assays, and similar results were observed (Fig. 2B–D).

### lncRNA *HAR1A* suppresses NSCLC growth and metastasis in vivo

**Fig. 3:**
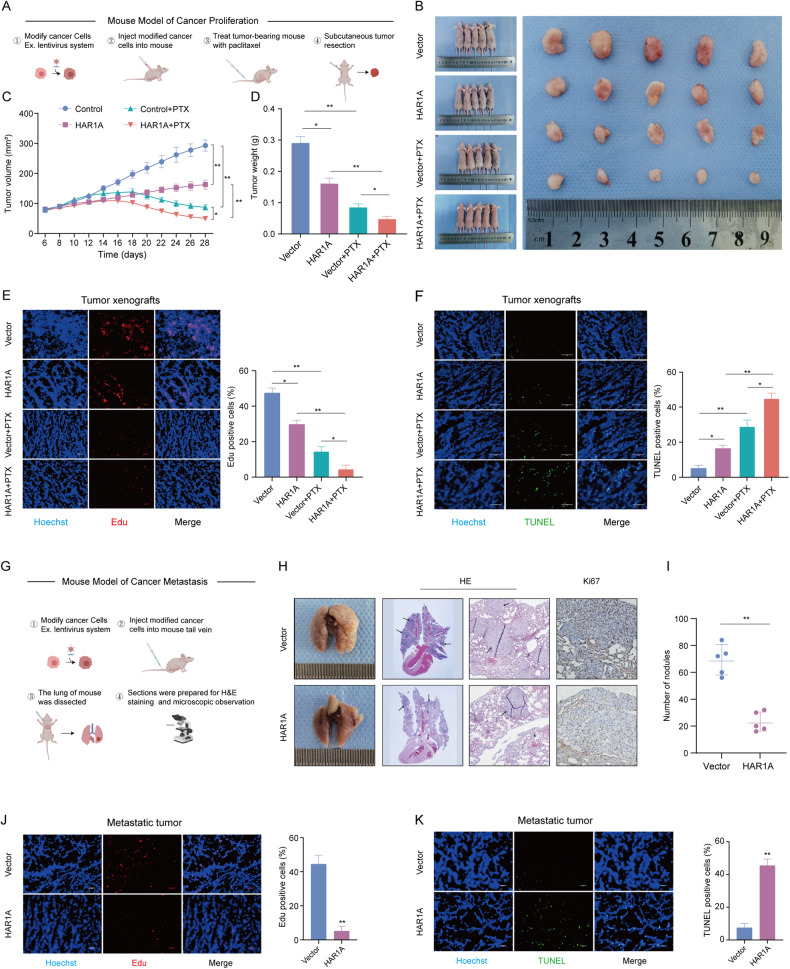
*HAR1A* enhances the cytotoxic effects of paclitaxel (PTX) on NSCLC cells and inhibits tumor metastasis in vivo. Impacts of PTX and *HAR1A* overexpression on tumor growth of PTX-treated xenograft mice. **A** The workflow of in vivo tumor growth study. The effect of PTX on the growth of tumors originated from stable cells infected with lentiviruses containing *HAR1A* cDNA (*HAR1A*) or empty lentivirus vectors (vector). Paclitaxel was administered at a dose of 15 mg/kg once every four days. **B** Representative images of mice bearing subcutaneous tumor xenografts for indicated groups and resected tumors from corresponding mice. **C** Tumor growth curves (volume) in mice subcutaneously injected with LV-*HAR1A* or LV-vector stably infected H1299 cells with or without PTX inventions. **D** Tumor weight in mice injected with H1299 stable LV-*HAR1A* or LV-vector cells at day 28 after PTX treatment. **E**, **F** EdU and TUNEL assays were performed to detect proliferation and apoptosis in metastatic nodes, respectively, followed by quantifications. **G** The workflow of in vivo tumor metastasis study. **H**, **I** Ex vivo mouse lungs elucidated that reduced lung metastasis in mice injected with *HAR1A*-overexpressing NSCLC cells, compared with mice receiving vector cells. HE and Ki67 stains showed metastatic nodes and proliferating cells. **J**, **K** EdU and TUNEL assays on metastatic nodes and quantifications. *p < 0.05; **p < 0.01; ***p < 0.001; ****p < 0.0001.

Given our in vitro findings, we subcutaneously implanted stable *HAR1A*-overexpressing H1299 cells into female BALB/c nude mice to assess the effects of *HAR1A* and PTX tumor growth (Fig. 3A). We found that paclitaxel treatment or *HAR1A* overexpression significantly decreased tumor volume and weight compared to the control group (Fig. 3B–D). Paclitaxel administration further shrank tumor xenografts arising from *HAR1A*-overexpressing H1299 cells (Fig. 3B–D). EdU assay confirmed fewer Edu-positive cells in tumors with paclitaxel treatment or *HAR1A* overexpression alone and the least proliferating cells in the combination group, compared with the control group (Fig. 3E). TUNEL assay also verified more apoptotic cells in the combination group than others (Fig. 3F). These results suggest that paclitaxel and overexpression of *HAR1A* synergistically inhibited tumor growth. We next investigated whether *HAR1A* regulated tumor metastasis using a tail vein injection model (Fig. 3G). Five weeks post-injection, the lungs were excised, and gross analysis demonstrated that the lung surface was occupied by many metastatic tumor nodules in the control group, compared to relatively smooth surface lung morphology in the mice injected with cells stably expressing *HAR1A* (Fig. 3H). HE and IHC stains indicated tumor nodules in the lung and Ki67^+^ cells in the nodules (Fig. 3H). *HAR1A*-overexpressing H1299 cells led to significantly fewer nodules in the lung of host mice than vector-expressing cells (Fig. 3I). Moreover, EdU and TUNEL assays revealed significantly few EdU^+^ proliferative cells and more TUNEL^+^ apoptotic cells in metastasis nodules from stable *HAR1A*-expressing cells-injected mice than control mice (Fig. 3J, K).

### lncRNA *HAR1A* interacts with the oncogenic protein ANXA2

**Fig. 4:**
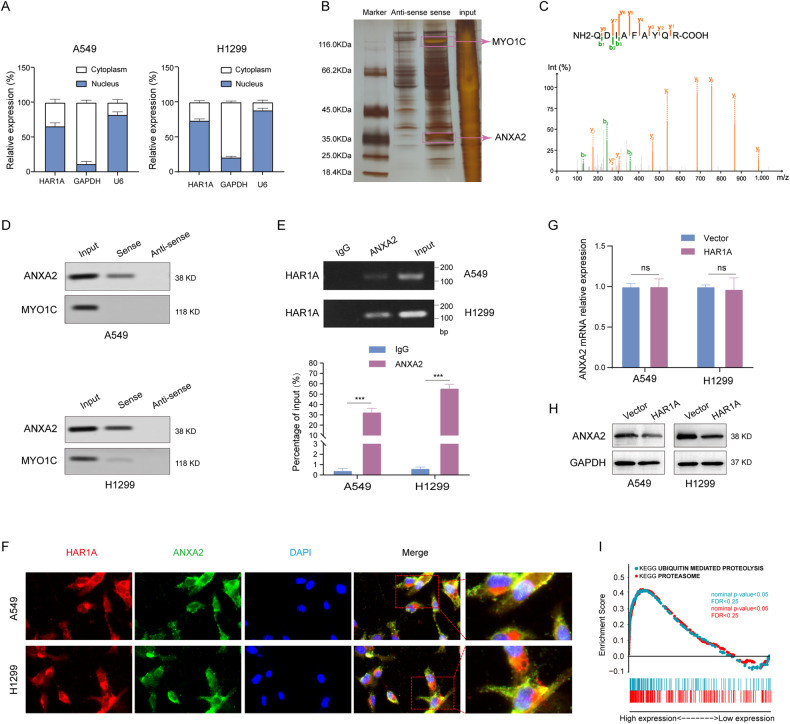
*HAR1A* interacts with the oncogenic protein ANXA2. **A** RT-qPCR was performed to quantify the mRNA levels of *HAR1A*, GAPDH, and U6 in nuclear and cytoplasmic fractions of A549 and H1299 cells. **B** RNA pull-downs were performed using biotinylated antisense and sense sequences of *HAR1A*. Co-precipitated proteins were resolved in SDS-PAGE and visualized with silver staining. **C** Identification of ANAX2 in precipitants of RNA pull-down assay by LC/MS. **D** Following the *HAR1A* RNA pull-down assay, western blot results showed the presence of ANXA2 among the pulled-down proteins. **E** RIP analysis with anti-ANXA2 antibody was conducted in A549 and H1299 cells to show co-precipitated *HAR1A* using RT-qPCR. **F** The representative photograph showing fluorescence in situ hybridization (FISH) with a probe against *HAR1A* and immunofluorescent staining with anti-ANXA2 antibody in A549 and 1299 cells. Red, green, and blue represent biotin-labeled probe against *HAR1A*, immunofluorescent staining of ANXA2, and DAPI staining of the nucleus, respectively. Yellow in the merged image indicates the colocalization of *HAR1A* and ANXA2 in the cells. **G** RT-qPCR analysis of the ANXA2 mRNA levels in LV-vector and LV-*HAR1A* treated A549 and H1299 cells. **H** Immunoblotting to examine ANXA2 levels in A549 and H1299 stable cells overexpressing *HAR1A* or control cells expressing LV-vector. **I** Gene set enrichment analysis (GSEA) to explore the downstream pathway of *HAR1A*. *p < 0.05; **p < 0.01; ***p < 0.001; ****p < 0.0001.

We next questioned how *HAR1A* inhibits NSCLC growth and metastasis. RT-qPCR results showed that *HAR1A* was mainly localized in the cell nucleus, but a small amount of this lncRNA was also seen in the cytoplasm (Fig. 4A). RNA pull-down assays, followed by mass spectrometry, were performed to identify candidate proteins interacting with *HAR1A*. Numerous *HAR1A*-binding proteins were enriched and immunoprecipitated (Fig. 4B). Among them, myosin IC (MYO1C) and annexin 2 (ANXA2) were found to be the most abundant protein among all identified proteins (Table S2), and they have been reported to be associated with carcinogenesis [22–24]. The peptide mass fingerprinting of ANXA2 is displayed in Fig. 4C. When we used *HAR1A* probe to perform RNA pull-down experiments, ANXA2 could be readily detected in the precipitates by western blotting in both cell lines, while only a weak band was observed for MYO1C in H1299 cells (Fig. 4D). RIP experiments using anti-ANXA2 antibodies were performed to validate the interaction between *HAR1A* and ANXA2 in the whole lysis of A549 and H1299 cells. As shown in Fig. 4E, RT-qPCR could amplify *HAR1A* in ANXA2 immunoprecipitates. FISH and immunocytostaining validated the colocalization of ANXA2 in two NSCLC cell lines (Fig. 4F). When we examined whether *HAR1A* regulated ANXA2 expression to transmit signaling, we found that ectopic expression of *HAR1A* did not significantly alter mRNA levels but reduced protein levels of ANXA2 (Fig. 4G, H). Interestingly, GSEA suggested that *HAR1A*-related genes are greatly enriched in the ubiquitin-mediated proteolysis and proteasome pathways (Fig. 4I), implying the potential roles of *HAR1A* in the ubiquitination and degradation of proteins.

### *HAR1A* facilitates TRIM65-mediated ANXA2 ubiquitination and degradation

**Fig. 5:**
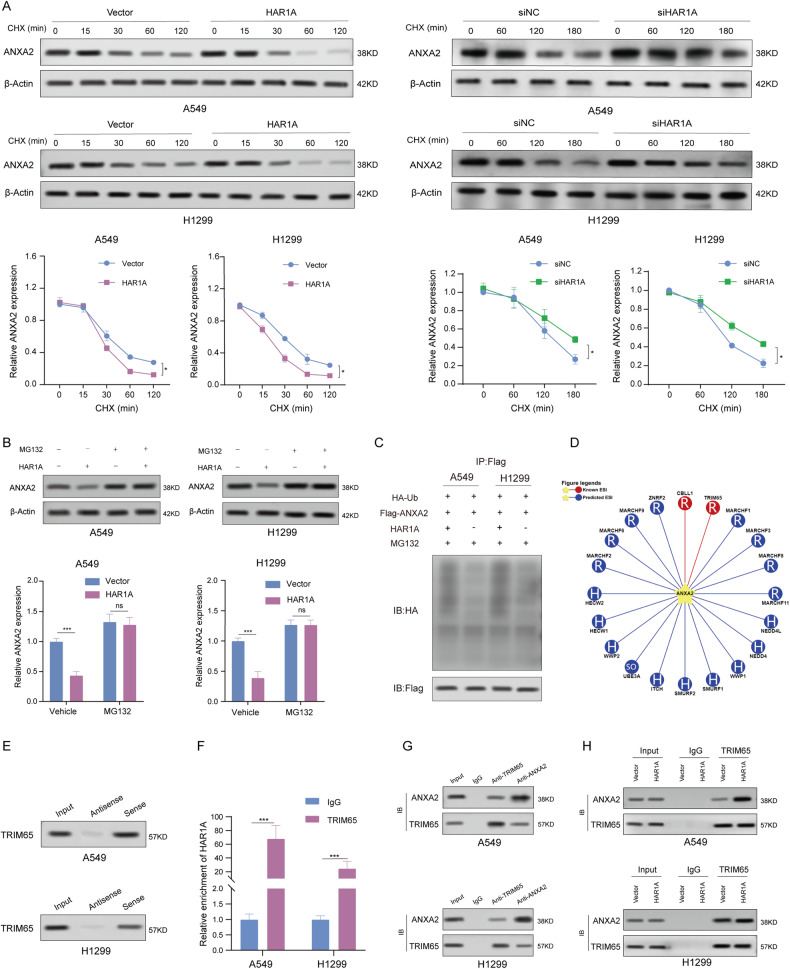
E3 TRIM65 mediates the ubiquitination and degradation of ANXA2. **A** Cells transfected with siNC or si-*HAR1A*, as well as cells with stable *HAR1A* overexpression, were treated with cycloheximide (CHX) for the specified periods. Subsequently, cell lysates were analyzed by western blot to examine the half-life of ANXA2 protein. Protein band intensities were quantified using Image J software. **B** Western blot was performed to check the effects of proteasome inhibitor MG132 on the ANXA2 expression levels. **C** HA-Ub and Flag-ANXA2 plasmids were co-introduced into stable A549 and H1299 LV-vector and LV-*HAR1A* cells. Cells were treated with MG132. Immunoprecipitation was performed with cell lysates using an anti-Flag antibody and western blot followed with an anti-Flag or anti-ubiquitin antibody. **D** Predicated E3 ligases for ANAX2 using the UbiBrowser tool. **E** *HAR1A* RNA pull-down assay and western blot showed the interaction between *HAR1A* and TRIM65. **F** RIP analysis coupled with RT-qPCR indicated that TRIM65 could precipitate *HAR1A* in A549 and H1299 cells. **G** Co-immunoprecipitation (Co-IP) of ANXA2 and TRIM65 in A549 and H1299 cells. Western blot with anti-ANXA2 antibody was used to probe the ANXA2 in the anti-TRIM65 antibody-mediated immunoprecipitates. Reverse co-IP was also performed to testify the immunoprecipitation of TRIM65 with anti-ANXA2 antibody. **H** Co-IP revealed that *HAR1A* facilitated the interaction between ANXA2 and TRIM65. *p < 0.05; **p < 0.01; ***p < 0.001; ****p < 0.0001.

The results of the cycloheximide (CHX) chase assay demonstrated that ANXA2 in *HAR1A* overexpression cells exhibited a shorter half-life when compared with control cells, whereas the half-life of ANXA2 was much longer in *HAR1A*-knockdown cells than in the control cells (Fig. 5A). Moreover, MG132 could block *HAR1A*-mediated ANXA2 degradation (Fig. 5B). Finally, we found that transfection of *HAR1A* enhanced the ubiquitination levels of ANXA2 (Fig. 5C). These results suggest that *HAR1A* may promote ANXA2 degradation by upregulating its ubiquitination levels. Using the online tool UbiBrowser^2.0^ (http://ubibrowser.bio-it.cn/ubibrowser_v3/), we retrieved 20 E3 ligases potentially mediating ubiquitination modification of ANXA2, such as MARCHF2, WWP2, NEDD4L, CBLL1, and TRIM65 (Fig. 5D). Among them, tripartite motif containing 65 (TRIM65) was reported to ubiquitylate ANXA2 in urothelial carcinoma of the bladder (UCB) [25]. Furthermore, of the predicted 20 E3 ubiquitin ligases, only TRIM65 was identified in the mass spectrometry results. Consequently, we examined whether *HAR1A* functions as a scaffold to facilitate interactions between ANXA2 and TRIM65, thereby regulating ANXA2 stability. We then investigated whether TRIM65 mediates ubiquitination and degradation of ANXA2 and whether *HAR1A* is involved in this process. First, using an RNA pull-down assay, we showed that *HAR1A* binds to TRIM65 in A549 and H1299 cells (Fig. 5E). We also verified the interaction through RIP in the two cell lines (Fig. 5F). Second, ANXA2 could be immunoprecipitated using an anti-TRIM65 antibody, while we further confirmed that TRIM65 was physically co-immunoprecipitated with ANXA2 using an anti-ANXA2 antibody (Fig. 5G). Ultimately, we also investigated the effects of *HAR1*A on TRIM65-ANXA2 interaction. Co-IP experiments revealed that anti-TRIM65 antibody enriched more ANXA2 in cells overexpressing *HAR1A* than in control cells (Fig. 5H), suggesting *HAR1A* overexpression boosted TRIM65’s interaction with ANXA2. These results indicated that *HAR1A* enhanced the interaction between ANXA2 and TRIM65 to facilitate TRIM65-mediated ANXA2 ubiquitination, consequently accelerating its degradation.

### *HAR1A* abrogates NSCLC cells’ aggressive behaviors by downregulating ANXA2

**Fig. 6:**
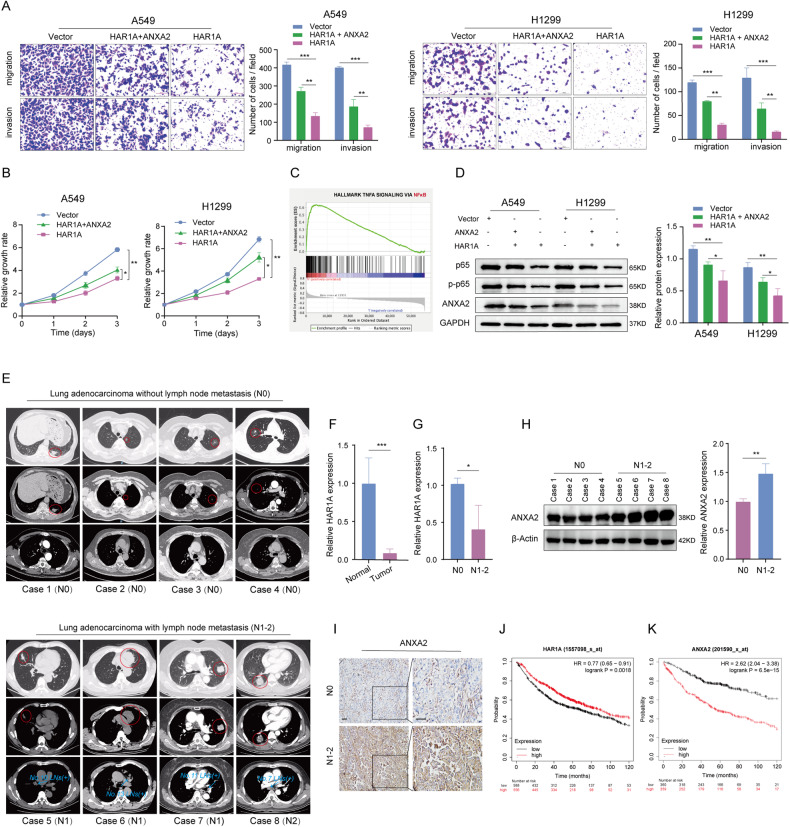
*HAR1A* diminished the malignant behaviors of A549 and H1299 cells by downregulating ANXA2. Stable A549 and H1299 LV-*HAR1A* cells were further transfected with ANXA2 plasmids. **A** Representative image of crystal violet-stained A549 and H1299 cells with modified expression of *HAR1A* and ANXA2 in the transwell assays. Quantification of migrated and invaded cells indicated that transfection of ANXA2 plasmids partially counteracted *HAR1A*-induced repression of tumor cell migration and invasion. **B** CCK8 was used to measure proliferation of A549 and H1299 cells transduced with LV-vector, LV- *HAR1A+*ANXA2 plasmid, and LV- *HAR1A+*empty plasmid. **C** Gene set enrichment analysis (GSEA) showed the downstream pathway of ANXA2. **D** Western blot to determine the protein levels of p65, p-p65, and ANXA2 in A549 and H1299 cells with indicated gene manipulations. Tumor samples were collected from eight NSCLC patients, along with matched normal tissues. **E** Chest computed tomography imaging of the eight NSCLC patients. The red circles highlight primary lung tumors and the blue arrows indicate regional lymph nodes with pathologically proven metastasis. According to the 8th edition of AJCC TNM staging, *N* category was defined as N0 (no metastatic lymph node), N1 (metastasis in ipsilateral peribronchial and/or ipsilateral hilar lymph nodes), N2 (metastasis in ipsilateral mediastinal and/or subcarinal lymph nodes). **F** RT-qPCR was used to measure *HAR1A* in normal tissues versus tumors. **G** Comparsion of *HAR1A* in N0 versus N1-2 tumors. **H** Western blot to compare the ANXA2 levels between N0 and N1-2 tumors. **I** ANXA2 immunohistochemistry staining in NSCLC tumor samples. **J**, **K** Kaplan–Meier survival curves for lung cancer patients dichotomized by the levels of *HAR1A* or ANXA2, using the K–M plotter. *p < 0.05; **p < 0.01; ***p < 0.001; ****p < 0.0001.

Since *HAR1A* decreases ANXA2 by shortening its half-life, we investigated whether ANXA2 is indispensable for *HAR1A*-induced inhibition on the proliferation and migration of A549 and H1299 cells. We first restored the expression of ANXA2 in stable *HAR1A*-overexpressing NSCLC cells by transfecting ANXA2 plasmids. We found that *HAR1A*-induced decreases in migration and invasion of NSCLC cells were partially recovered by the overexpression of ANXA2 (Fig. 6A). Moreover, CCK8 assay demonstrated that the re-expression of ANXA2 reversed *HAR1A*’s inhibitory effects on the proliferation of A549 and H1299 cells (Fig. 6B). These results substantiated that *HAR1A* suppresses the proliferation, migration, and invasion of cancer cells by reducing ANXA2 expression. Gene set enrichment analysis (GSEA) indicated that it may regulate NF-kB (Fig. 6C). Additionally, several publications have implied that ANXA2 functions by activating the NF-kB signaling pathway [18, 26, 27]. Our western blot results elucidated that *HAR1A* overexpression led to a reduction in the levels of p65 and its phosphorylated form; however, simultaneous re-expression of ANXA2 could reactivate the NF-kB signaling pathway suppressed by *HAR1A* (Fig. 6D). Since the oncogenic role of the NF-kB signaling pathway is well documented, it is biologically plausible to infer that *HAR1A* may exert tumor-suppressing functions by repressing the ANXA2/NF-kB axis. We also collected paired normal and tumor tissues from 8 patients with lymph node stage N0-2 NSCLC (Fig. 6E). RT-qPCR analysis showed a significant decrease of *HAR1A* in tumors versus normal tissues (Fig. 6F), and N1-2 tumors versus N0 tumors (Fig. 6G). Western blot results showed ANXA2 was significantly increased in N1-2 tumors compared with N0 tumors (Fig. 6H), which was also validated using immunohistochemistry staining (Fig. 6I). Moreover, K–M plotter indicated that high levels of *HAR1A* and ANXA2 in NSCLC were associated with OS in the opposite direction (Fig. 6J, K).

### METTL3-mediated m^6^A modifications expedites *HAR1A* disintegration

**Fig. 7:**
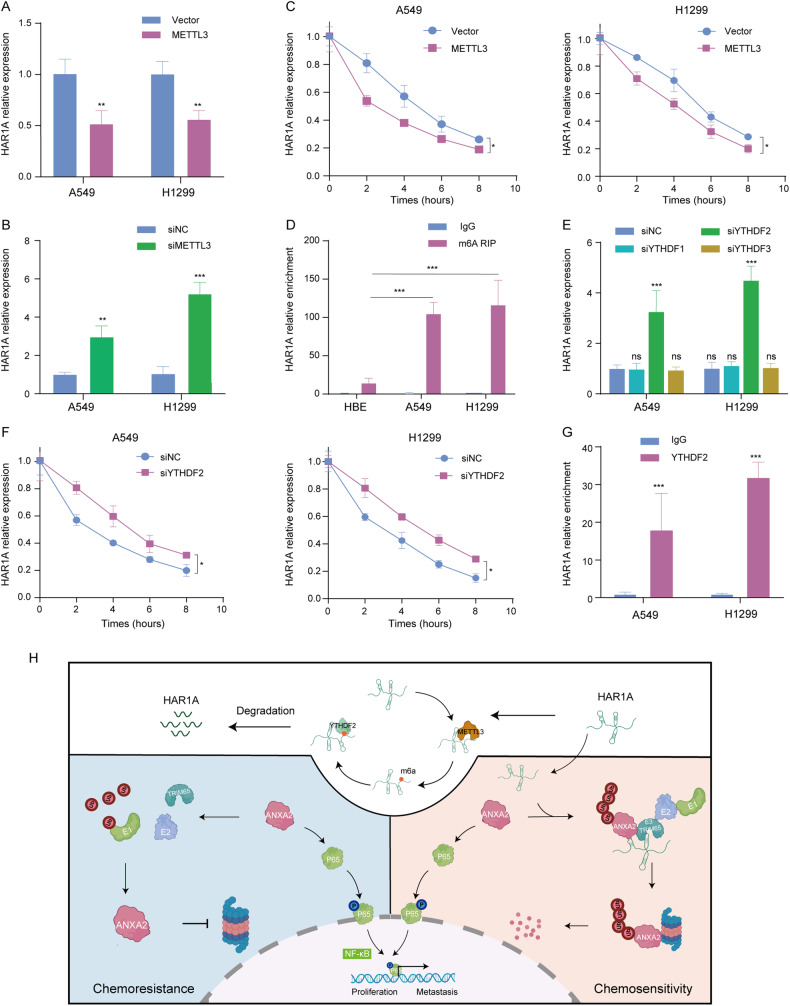
METTL3-mediated m^6^A modifications lead to *HAR1A* degradation. **A**, **B** RT-qPCR was performed to investigate the effects of overexpression and knockdown of METTL3 on the *HAR1A* levels in A549 and H1299 cells. **C** After inhibiting RNA synthesis with actinomycin D, *HAR1A* degraded faster in NSCLC cells with METTL3 overexpression than in control vector cells at different times. **D** m^6^A RIP coupled with RT-qPCR showed that *HAR1A* was subjected to m^6^A modification, and significantly more m^6^A-modified *HAR1A* RNAs were enriched in A549 and H1299 than in HBE cells. **E** Cells were transduced with siRNAs targeting YTHDF1, YTHDF2, YTHDF3, and scramble controls. siYTHDF2 treatment reduced *HAR1A* levels as shown by RT-qPCR. **F** In cells where RNA synthesis was blocked with actinomycin D, YTHDF2 siRNA slowed down the degradation of *HAR1A*. **G** RIP assay, followed by RT-qPCR, revealed the precipitation of YTHDF2 with *HAR1A*. **H** Schematic diagram of molecular mechanisms. *p < 0.05; **p < 0.01; ***p < 0.001; ****p < 0.0001.

Finally, we questioned how the expression levels of *HAR1A* declined in NSCLC compared with normal lung tissues. Dysregulation of RNA m^6^A modification has been well known to be implicated in carcinogenesis. Methyltransferase Like 3 (METTL3), installing m^6^A in RNAs, is highly expressed in NSCLC and promotes cancer progress [19–21]. Therefore, we scrutinized whether METTL3 regulates *HAR1A* expression. Both gain and loss function analysis showed that METTL3 negatively regulated *HAR1A* (Fig. 7A, B). Moreover, METTL3 overexpression speeded up *HAR1A* degradation with time (Fig. 7C), denoting that METTL3 might destabilize *HAR1A* through m^6^A modification. Indeed, meRIP-qRCR experiments showed that m^6^A-modified *HAR1A* was enhanced in A549 and H1299 cells in comparison to HBE cells (Fig. 7D). By screening the known m^6^A readers, including YTHDF1-3, we found that the knockdown of YTHDF2 leads to upregulation of *HAR1A* (Fig. 7E). Consistently, YTHDF2 depletion prevented *HAR1A* from degrading (Fig. 7F). Finally, RIP experiments using anti-YTHDF2 antibodies confirmed the binding of YTHDF2 to *HAR1A* (Fig. 7G).

## Discussion

Lung cancer is the top cause of cancer-related mortality. NSCLC is the most predominant type of lung cancer. Despite revolutionary advances in treatment modalities, including surgery, radiation, chemotherapy, targeted therapies, and immunotherapies, the prognosis of NSCLC remains unsatisfying. Due to the lack of reliable early-detection biomarkers, most patients could not be diagnosed until the development of locally advanced or widely metastatic tumors. Therefore, further dissecting the molecular landscape of tumors, particularly ncRNAs, has the promise of discovering novel cancer-related biomarkers for early diagnosis and prognosis prediction and novel molecular targets for developing efficacious therapeutic strategies.

As far as we know, only up to 2% of human genomic DNA is protein-coding, whereas ncRNAs may constitute 70% of the human genome. In contrast to limited protein, tons of functional lncRNAs warrant to be identified, which may have great potential to be biomarkers or druggable targets for cancer [6–8]. Tremendous lncRNAs are deregulated in cancers, and monitoring circulating lncRNA is minimally invasive [6]. For instance, lncRNA *H19* showed promise for being an early diagnosis marker of gastric cancer. The levels of plasma *H19* significantly declined post-surgery in patients with gastric cancer, indicating the potential application of *H19* in the early diagnosis and postoperative monitoring settings [28]. Furthermore, many strategies for developing ncRNA-targeting small-molecule compounds have emerged, such as small-molecule microarray, structure-based designing, phenotypic screening, and pharmacological validation approaches [8]. As a result, several small-molecule compounds, including bisphenol-A, mitoxantrone, and enoxacin have been shown to modulate or selectively target ncRNAs in different types of cancer [8]. To date, some ncRNA-regulating small-molecule compounds have been pharmacologically confirmed. Bisphenol-A (BPA) and diethylstilbestrol (DES) are typical examples. LncRNA *HOTAIR* was oncogenic in a variety of cancers. *HOTAIR*, which prompted the proliferation and migration of breast cancer cells, is transcriptionally activated by estradiol [29]. The same team also found that low concentrations of BPA and DES could significantly induce *HOTAIR* expression in breast cancer cells [30], suggesting that small-molecule compounds could regulate the levels of lncRNAs.

Other teams and we have shown that lncRNA *HAR1A* is a tumor suppressor in several cancers, including HCC [31], oral cancer [10], and NSCLC [9]. This study aimed to discover the molecular mechanisms underpinning *HAR1A*’s tumor-suppressing functions in NSCLC. We found that overexpression of *HAR1A* could reduce the proliferation, migration, invasion, and EMT of NSCLC cells. *HAR1A* strengthened the interaction between the oncogenic protein ANXA2 and its E3 ligase TRIM65, thereby promoting ubiquitination and degradation of ANXA2 to inactivate the NF-κB signaling pathway. Similarly, Lee et al. demonstrated that si-*HAR1A* treatment augmented proliferation, migration, and EMT in oral squamous cell carcinoma (OSCC) cells [10].

Shi et al. reported that loss of *HAR1A* in HBV-Induced HCC in Chinese Patients was related to inferior clinical outcomes [31]. Tumor-suppressive lncRNAs are very common. LncRNA *DRAIC* was downregulated in castration-resistant advanced PC. This lncRNA tumor suppressor inhibited tumor growth and invasion by inactivating nuclear factor-κB (NF-κB). lncRNA *DRAIC* binds to the IκB kinase complex (IKK) complex to inhibit the phosphorylation and proteasomal degradation of NF-κB inhibitor-α (IκBα), thereby suppressing the NF-κB signaling pathway [5]. LINC00261 was epigenetically silenced in LUAD, and its deficiency was validated in liver, breast, and gastric cancer. Enforced expression of LINC00261 inhibited migration and proliferation of LUAD cell lines through induction of a G2/M cell cycle arrest and activation of the DNA damage response [32].

The regulatory mechanisms of lncRNAs have been studied extensively, which interact with DNA, RNA, or proteins to regulate various cellular processes, including transcriptional regulation, chromatin structure, RNA stability, and cell proliferation. Wang and colleagues categorize the numerous functions of lncRNA into four archetypes of molecular mechanisms: signals, guides, scaffolds, and decoys; however, these mechanisms are not mutually exclusive [33]. First, lncRNAs can transduce signals because the transcription of single lncRNAs is tightly regulated in response to diverse stimuli. Moreover, lncRNAs do not require translation and respond more quickly than proteins regarding signal transduction. Second, lncRNAs can serve as molecular decoys. For instance, lncRNAs bind and sequester a protein target without exerting extra functions. In other words, lncRNA trapped RBPs (e.g., transcription factors, enzymes, or other regulatory factors) to not fulfill their tasks. LINC00941 promoted the progress of pancreatic cancer (PC) through the decoying mechanism. LINC00941 bound to ANXA2 as a decoy to competitively inhibit the interaction of ANXA2 with NEDD4L, an E3 ligase, consequently preventing ANXA2 from NEDD4L-mediated ubiquitination and proteasomal degradation. The upregulated ANXA2, in turn, activated FAK/AKT signaling to fuel PC cell proliferation and metastasis [34]. The third molecular mechanism of lncRNA is the guide-lncRNA interacts with and conveys protein to a specific location, especially in the case of regulating gene expression in *trans*. Finally, lncRNAs can provide a platform to facilitate intermolecular interactions since lncRNA contains different domains that bind more than one effector molecule. In the current study, *HAR1A* might act as a scaffold to aid the interaction between TRIM65 and ANXA2, destabilizing the latter to inactivate the NF-κB signaling pathway. In other words, *HAR1A* bonds TRIM65 and ANXA2 simultaneously to bring them together in both time and space. By doing so, *HAR1A* greatly enhanced the enzymatic activity of TRIM65 to attach ubiquitins to ANXA2 for subsequent proteasomal degradation. The future exhaustive understanding of how these molecules are assembled and regulated may enable therapeutic strategies to target signaling components and reshape cellular behavior precisely.

ANXA2 is a multi-functional protein and participates in various cellular processes involving signal transduction, endocytosis, cytokinesis, actin remodeling, mRNA translocation, and DNA repair. Accumulating evidence has shown the contribution of Annexin 2 in the development of lung cancer. Coculture with cancer-associated fibroblasts (CAFs) led to acquired resistance of NSCLC cells to tyrosine kinase inhibitors (TKIs). The underpinning mechanism was that growth factors HGF and IGF-1 released by CAFs upregulated ANXA2 and increased its phosphorylation, thereby inducing EMT of NSCLC cells [35]. ANXA2 interacted with T-cell immunoglobulin and mucin domain-containing molecule 4 (TIM-4) and mediated TIM-4-induced lung cancer progression by activating PI3K/AKT/OPA1 axis [36]. Ubiquitination is a critical post-translational modification to control ANXA2 activity in carcinogenesis, and it is subjected to ubiquitination-mediated degradation [16]. E3 ubiquitin ligase FBXW7 facilitated the ubiquitination and subsequent disintegration of ANXA2. Loss of FBXW7 promotes esophageal squamous cell carcinoma (ESCC) progression by enhancing ANXA2 to activate the MAPK pathway [16]. Moreover, Wei et al. showed that another E3 enzyme, TRIM65, installed ubiquitin in amino acid residues of ANXA2 in urothelial carcinoma of the bladder [25]. Therefore, decreased *HAR1A* in NSCLC potentiates cancer growth and metastasis by upregulating the ANXA2.

We also investigated the effector signaling pathway downstream of ANXA2. Several studies disclosed the direct interaction of ANXA2 with p65 [17, 18, 37]. Trim33 promoted ANXA2’s interaction with p50/p65 subunits of NF-κB by catalyzing lysine 63 (K63)-linked ubiquitination of Annexin A2 (Anxa2) to stir inflammation response in psoriasis [18]. In the LUAD, LINC01614 formed a trimeric complex with ANXA2 and p65 to facilitate the latter two molecules’ interaction and the activation of NF-κB, eventually elevating cancer cell growth [17]. Consistently, we demonstrated that *HAR1A* overexpression lowered the activity of the NF-κB pathway by downregulating ANXA2. Therefore, decreased *HAR1A* in NSCLC potentiates cancer growth and metastasis by upregulating the ANXA2/NF-κB axis.

Finally, our results demonstrated that METTL3 suppressed ANXA2 levels and reduced stability. It turned out that YTHDF2 recognized the m^6^A modification in *HAR1A* to accelerate its degradation. Dysregulation of RNA m^6^A modification has been well known to be implicated in carcinogenesis. Methyltransferase Like 3 (METTL3), installing m^6^A in RNAs, is highly expressed in NSCLC and promotes cancer progress [19–21]. Apart from mRNAs, lncRNAs can also be regulated by m^6^A modification. Many studies demonstrated that YTHDF2 mediated the stability of m^6^A-modified RNAs in a cotext-dependent manner [38–41]. LINC02038, subjected to METTL3-mediated m6A modification, was a tumor suppressor and decreased in CRC. YTHDF2 was shown to recognize m6A modification in LINC02038 and trigger its degradation [38]. Consistently, Ye et al. reported that YTHDF2 sensed m^6^A modifications in the tumor-suppressive lncRNA *CARMN* and enabled m^6^A-caused breakdown of this lncRNA in cervical cancer [41]. Another study reported that METTL3-mediated m6A modification could stabilize LINC00839, an oncogenic lncRNA, in a YTHDF2-dependent manner. The upregulated LINC00839 was associated with glioblastoma progression and radiation resistance by activating Wnt/β-catenin activation [39].

In conclusion, our results indicated that *HAR1A* deficiency disables the ubiquitination and proteasomal degradation of ANXA2 to activate the NF-κB pathway, leading to lung cancer progress. The METTL3- YTHDF2 axis partially interprets the loss of *HAR1A* in NSCLC.

## Materials and methods

### Clinical samples

The NSCLC and matched adjacent normal tissues were collected from August 2022 to October 2022 in the Harbin Medical Cancer Hospital. Fresh tissue samples were snap-frozen in liquid nitrogen and stored in a deep freezer (−80 °C) until usage. The Ethical Committee of the Harbin Medical Cancer Hospital approved the study. Written informed consent was obtained by all participants in the study.

### Cell culture

Normal human lung bronchial epithelial cell lines (HBE) and NSCLC cell lines (H1299, A549, H1650, and H1993) were purchased from the Cell Bank of Type Culture Collection of Chinese Academy of Sciences (CBTCCCAS, Shanghai, China). All the cells were cultured in DMEM (Corning, USA) with the addition of 10% fetal calf serum and 10% penicillin/streptomycin (Gibco, USA). The cultures were maintained in an incubator preset at 37 °C in a 5% CO_2_ humid environment.

### Transfection

Both lentiviruses expressing *HAR1A* (LV-*HAR1A*) and control vectors (LV-vector) were purchased from the Genechem company (Shanghai, China). The full length of human *HAR1A* cDNA was directly synthesized to construct the *HAR1A* overexpression vector. *HAR1A* overexpression lentiviral vector GV717 (CMV enhancer-MCS-sv40-puromycin) and control lentiviral vector were constructed and packed using 293T cells. Stable cell lines overexpressing *HAR1A* or vector were established by lentiviral infection followed by puromycin (Calbiochem, USA) selection. Then, *HAR1A* expression was evaluated by quantitative PCR (qPCR). The knockdown of *HAR1A* was achieved by transfection of siRNA, specifically targeting the lncRNA (General Biol, China).

Short interference RNAs (siRNAs) against human METTL3 (siMETTL3), YTHDF1 (siYTHDF1), YTHDF2 (siYTHDF2), YTHDF3 (siYTHDF3) and matched negative controls (siNC) were provided by Genechem (Shanghai, China). Plasmids overexpressing METTL3, ANXA2, and respective controls (Ctrl) were cloned into the pcDNA3.1 vector (Genechem, China). The transfections were performed with INTERFERin® (Polyplus-transfection® SA) according to the manufacturer’s instructions. The target sequences used for the siRNAs are listed in Table S1.

### Reverse transcription and quantitative (RT-qPCR)

TRIZOL reagent (Invitrogen) was used to extract total RNA. First-strand cDNA was prepared with the Transcriptor First-Strand cDNA Synthesis Kit (Roche, USA). Real-time PCR was performed using FastStart Universal SYBR Green Master (ROX) (Roche) on a 7500 Fast Real-Time PCR system (ABI, USA). The relative expression of *HAR1A*, Vimentin, N-cadherin, E-cadherin, ANXA2, U6, and GAPDH was detected at least in triplicate with indicated primers (Table S1). The melting-curve analyses were used to confirm PCR product specificity. Relative expression of genes of interest was determined using the 2^–ΔΔCt^ method and normalized to GAPDH.

### Western blot

The harvested cells were pelleted and lysed in ice-cold RIPA buffer with 0.1 M PMSF. Protein concentrations of samples were quantified by using the BCA Protein Assay Kit. Protein samples (30 µg) were separated by polyacrylamide electrophoresis, transferred onto polyvinylidene fluoride (PVDF) membranes and incubated with specific antibodies. The primary detection antibodies that were used in the study are listed in Table S1.

### Subcellular fractionation

Cytoplasmic & Nuclear RNA Purification Kit (NORGEN BIOTEK CORP. Thorold, ON, Canada) was used to extract RNA from nuclear and cytoplasmic fractions of A549 and H1299 cells according to the manufacturer’s instructions. GAPDH and U6 exclusively expressed in nuclear and cytoplasm, respectively, were used as positive controls for *HAR1A* in the qRT-PCR assay.

### Cell counting kit (CCK8) assay

Cell proliferation was assessed using a CCK8 kit (Dojindo, Japan) according to the manufacturer’s instructions. A total of 4 × 10^3^ cells were seeded into 96-well plates and cultured at 37 °C. Medium containing 10% CCK8 replaced the original medium and incubated at 37 °C for 2 h, and the absorbance was finally determined at 450 nm using a microplate reader. For paclitaxel sensitivity, the cells were treated with different concentrations of paclitaxel for 24 h. After treatment, the media were removed, and absorbance was then measured at 450 nm.

### Colony formation assay

In total, 600 cells were seeded in 6-well plates and cultured in a complete medium supplemented with 10% FBS. After 14 days, the cells were washed with PBS, fixed in methanol for 30 min, and stained with 0.1% crystal violet dye. Finally, the number of colonies was counted by three different individuals.

### EdU assay

Cells were incubated with 100 μM EdU (Ribobio, Guangzhou, China) for 3 h at 37 °C and then fixed in 4% paraformaldehyde for 30 min. After that, the cells were permeabilized with 0.4% Triton X-100 for 10 min and incubated with Apollo® reagent (100 μl) for 30 min. Finally, the cells were stained with Hoechst for 30 min. The images were observed under an inverted fluorescence microscope. The cell proliferation rate was calculated using the ratio of EdU-positive cells (red) to Hoechst-positive cells (blue).

### TUNEL assay

Apoptotic cells were identified by terminal deoxynucleotidyl transferase-mediated dUTP nick end-labeling (TUNEL) staining using the In Situ Cell Death Detection Kit (11684817910, Roche). The sections were analyzed by fluorescence microscopy (Olympus, Japan). The average number of TUNEL-positive cells in three images from each treatment group was calculated.

### Tumor cell migration and invasion assays

Both cell migration and invasion experiments were conducted with Transwell polycarbonate membrane cell culture inserts (BD Biosciences, CA, USA). A total of 3 × 10^4^ cells were added to the upper chamber, and the cells were allowed to migrate to the lower chamber for 24 h. (Matrigel was applied for the filter for invasion assay (BD Biosciences, CA, USA). The migrated/invaded cells were fixed with 4% paraformaldehyde and stained with 0.1% crystal violet staining for 30 min. The cells were counted in three independent fields under an optical microscope to calculate the transwell capacity.

### Wound healing assay

Cells were seeded to 6‐well plate and incubated until approximately 90% confluence. A plastic tip was used to create a wound at the cell surface. Then, PBS buffer was used to remove dissociated cellular fragments. The speed of wound healing was observed and imaged using a microscope, and the closure rate was estimated. Experiments were repeated in triplicates.

### Immunofluorescence staining

The cells were fixed with 4% paraformaldehyde for 30 min at room temperature and then permeabilized with 0.1% Triton X-100 for 15 min. After blocking with 5% BSA, the cells were incubated with the primary antibodies E-cadherin (ab40772, Abcam, 1:500), N-cadherin (ab18203, Abcam, 1:200), and Vimentin (ab92547, Abcam, 1:250) at 4 °C overnight. The next day, the cells were washed and incubated with secondary antibodies for 1 h at room temperature. Subsequently, cells were washed, and nuclei were counterstained with DAPI (Beyotime Company, China). All images were acquired using an inverted fluorescence microscope.

### Animal models

For in vivo experiments, H1299 cell lines stably transfected with negative control (NC) and *HAR1A* expression (*HAR1A*) were first established. BALB/c nude mice (5 weeks old) were obtained from Beijing Vital River Laboratory Animal Technology Co, Ltd (Beijing, China). For the proliferation model, 20 nude mice were randomly assigned to four groups, and mice were injected subcutaneously with 1 ml of cell suspension (2 × 10^6^ cells/mouse). After the tumors grew to approximately 100 mm^3^ in size, 15 mg/kg paclitaxel was administered once every four days for a total of three courses. Tumor volume was measured once every two days by using calipers at the indicated time points. The tumor volume was estimated by the following formula: volume (mm^3^) = 0.5 × length (mm) × width^2^ (mm^2^). The whole body weight of mice was measured once every two days as indicated. For the metastasis model, 1 × 10^6^ cells were injected intravenously. Five weeks later, mice were sacrificed, and lungs were collected and fixed in 4% formaldehyde for H&E and immunofluorescent (IF) staining, respectively. The number of metastatic nodes in lung tissues was counted in five mice in each group. The Institutional Animal Care and Use Committee of the Center of Harbin Medical University approved the protocol.

### RNA pull-down assay

*HAR1A* RAN pull-down assay was conducted using the Pierce™ Magnetic RNA-Protein Pull-Down (Thermo Scientific, USA) and MAXIscript® (Ambion, USA) kits based on protocols provided by manufacturers. Cells lysed in RIPA buffer were incubated with in vitro transcribed full-length *HAR1A* probes and antisense probes labeled by biotins (Gene Create, China). Briefly, NSCLC cell extracts were incubated with biotinylated *HAR1A* or antisense-*HAR1A* (control), affinity precipitated using streptavidin-conjugated beads. The RNA-binding protein complexes were collected, washed, eluted, and analyzed by Liquid Chromatography and Mass Spectrometry (LC/MS) and western blot.

### Shotgun analysis

RNA pull-down samples were resolved in SDS-PAGE and stained with Bio-Safe Coomassie (Bio-Rad). Gels with protein bands were excised and minced into small pieces, followed by overnight digestion at 37OC. Peptides retrieved from the gel were dried and resuspended in a loading buffer. The Thermo Scientific UltiMate 3000 RSLCnano system combined with Thermo Scientific Q Exactive HF-X hybrid quadrupole-Orbitrap mass spectrometer was adopted to analyze peptides. Data-dependent (DDA) acquisition mode was applied to obtain the tandem mass spectrometry (MS/MS) data. We processed raw MS data using MaxQuant software (V1.6.6) using Andromeda, a peptide search engine based on a probabilistic scoring model. Protein sequences retrieved from the complete human proteome of Uniprot (Dec 2021, 20381 protein sequences) were utilized to determine the protein identities. The relative quantitative analysis was performed by calculating iBAQ values for identified proteins.

### RNA immunoprecipitation (RIP)

Cells were lysed with lysis buffer containing protease and RNase inhibitors. The lysates were centrifuged at 15,000 × *g* for 10 min, and the resulting supernatants were used to perform RIP assay with the Magna RIP™ RNA-Binding Protein Immunoprecipitation Kit (Millipore, USA). Antibodies used for immunoprecipitation include primary antibodies against ANXA2, TRIM65, YTHDF2, m6A, and IgG. The precipitated complexes were then washed, and the RNA was isolated using RNAiso Plus (TAKARA) for subsequent qRT-PCR analysis.

### RNA fluorescence in situ hybridization (FISH)

For FISH experiments, cells were grown on coverslips and fixed with 4% paraformaldehyde (Sigma-Aldrich, Germany) for 15 min. Subsequently, permeabilization was performed using 0.1% Triton X-100 (BioFroxx, Guangzhou, China) for 10 min. Following that, sections were treated with 20 μg/mL protease K (Servicebio, China) at 37 °C for 5 min. The samples were then hybridized overnight with 8 ng/μL m-*HAR1A* probe (5′-CY3-CGGACCGGAGGGAGAGCCGGGCGCAGAGACCGAGGCACA-CY3-3′) (Gene Pharma, China) at 37 °C, and subsequently incubated overnight at 4 °C with anti-ANXA2 (#8235, 1:100; CST, USA). After three PBS washes, cells were incubated with the secondary antibody (BosterBio, BA1105, China) for 1 h at room temperature in the dark. Nuclei were stained with DAPI (Servicebio) for 5 min. Finally, the slices were sealed with anti-fluorescence quenching tablets. Confocal microscopy (LSM880, Carl Zeiss) was used to capture all microscopic images.

### Protein degradation assay

Cycloheximide (CHX) chase assay was used to determine ANXA2 protein stability. A549 and H1299 cells overexpressing *HAR1A* or control vector cells were seeded in the cell culture plates. After 24 h, CHX was added to the culture media at a final concentration of 200 μg/mL to halt de novo protein synthesis. Cells were collected at specified time points after CHX treatment, and the protein levels of ANXA2 and β-Actin were assessed using western blotting.

### Ubiquitination assay

Ubiquitination assays were performed in LUAD cells with either control vectors or *HAR1A* overexpression. These cells were transfected with pcDNA3.1-HA-ubiquitin (HA-Ub) and Flag-ANXA2 plasmids. After 24 h of transfection, cells were treated with 50 μg/mL MG132 for 8 h, followed by lysis with ice-cold RIPA buffer (P0013C, Beyotime, China). The proteins in the cell lysate were immunoprecipitated using an anti-Flag antibody to enrich ubiquitinated ANXA2. The resulting immunoprecipitates were subjected to western blot analysis to determine ANXA2 protein and its ubiquitination levels using anti-HA and anti-Flag antibodies.

### Co-immunoprecipitation (Co-IP)

Whole-cell lysates prepared with IP lysis buffer were used for endogenous IP. The supernatants were incubated with primary antibodies against TRIM65, ANXA2, or normal mouse/rabbit IgG. The antibody-protein complexes were then incubated with Protein A/G PLUS-Agarose (Santa Cruz Biotechnology). The agarose-antibody-protein complexes were collected using a magnetic separator and analyzed by western blot with corresponding antibodies.

### RNA stability assay

The RNA stability assay was conducted following the previously described method (PMID: 31230592). A549 and H1299 cells were cultured in 6-well plates overnight. Actinomycin D (Med-ChemExpress) was added to inhibit gene transcription at a concentration of 5 μg/mL. The total RNA was subsequently extracted from harvested cells at different time points, and levels were quantified using RT-qPCR. The RNA levels in the indicated groups at different time points were calculated and normalized to GAPDH.

### Statistical methods

Data are presented as the mean ± standard deviation of at least three independent experiments for each cellular experimental group and at least five independent experiments for each animal group. Between-group differences concerning continuous variables were assessed using Student’s *t*-test or one-way/two-way ANOVA whenever appropriate. *P* values < 0.05 were indicative of statistical significance. Statistical analyses were performed using SPSS 17.0 software and GraphPad Prism 8 software.

### Supplementary information

Merge supplementary material file

Table S1

Table S2

Supplemental Figure 1

Supplemental Figure 2

Figure legends

## Data Availability

The data sets in this study are available from the corresponding author upon reasonable request.
