# Supplementary material for: METTL3-mediated deficiency of lncRNA HAR1A drives non-small cell lung cancer growth and metastasis by promoting ANXA2 stabilization
Source: Cell Death Discov. 2024 Apr 30;10:203. doi: 10.1038/s41420-024-01965-w (PMC11061277; doi:10.1038/s41420-024-01965-w)
Supplement: Supplementary file 2 — Table S1 [file 41420_2024_1965_MOESM2_ESM.docx]

**Table S1: information about the RNA interfering, sequences of RT-qPCR primers and antibodies used for WB.**

**information about the RNA interfering**

| **Name** | **Sequences of siRNA** |
| --- | --- |
| siNC | Sense 5'-UUCUCCGAACGUGUCACGUTT-3' |
|  | Antisense 5'-ACGUGACACGUUCGGAGAATT-3' |
| siHAR1A-1 | Sense 5'-GUGUGAAUGGAGUAUGAAUTT-3' |
|  | Antisense 5'-AUUCAUACUCCAUUCACACTT-3' |
| siHAR1A-2 | Sense 5'-GGAAAUGGUUUCUAUCAAATT-3' |
|  | Antisense 5'-UUUGAUAGAAACCAUUUCCTT-3' |
| siHAR1A-3 | Sense 5'-GUGAAAUGCCUCAUGAACUTT-3' |
|  | Antisense 5'-AGUUCAUGAGGCAUUUCACTT-3' |
| siMETTL3 | Sense 5′- CTGCAAGTATGTTCACTATGA -3 |
| siYTHDF1 | Sense 5′-GAACAAAAGGACAAGAUAAUA-3′ |
|  | Antisense 5′-CAAAAGGACAAGAUAAUAAAG-3′ |
| siYTHDF2 | Sense 5′-GCACAGAAGUUGCAAGCAAUG-3′ |
|  | Antisense 5′-UUGCUUGCAACUUCUGUGCUA-3′ |
| siYTHDF3 | Sense 5′-AGAUGGUGUAUUUAGUCAACC-3′ |
|  | Antisense 5′-UUGACUAAAUACACCAUCUGG-3′ |

**Sequences of RT-qPCR primers.**

| **Name** | **Primer sequence (5’-3’)** |
| --- | --- |
| GAPDH (Forward) | ACAGCCTCAAGATCATCAGC |
| GAPDH (Reverse) | GGTCATGAGTCCTTCCACGAT |
| HAR1A(Forward) | ACTCTGGTGTGTCCCGTTTGAA |
| HAR1A (Reverse) | TCTGTGTGTTGCCACCTCCG |
| U6 (Forward) | GGAACGATACAGAGAAGATTAGC |
| U6 (Reverse) | TGGAACGCTTCACGAATTTGCG |
| ANXA2(Forward) | GAGCGGGATGCTTTGAACATT |
| ANXA2(Reverse) | TAGGCGAAGGCAATATCCTGT |
| E-Cadherin (Forward) | AACAGGATGGCTGAAGGTGA |
| E-Cadherin (Reverse) | CCTTCCATGACAGACCCCTT |
| N-Cadherin(Forward) | ATATTTCCATCCTGCGCGTG |
| N-Cadherin(Reverse) | GTTTGGCCTGGCGTTCTTTA |
| Vimentin(Forward) | GGACCAGCTAACCAACGACA |
| Vimentin(Reverse) | AAGGTCAAGACGTGCCAGAG |

**Protein antibodies used for Western blotting**

| **Antibody** | **Manufacturer** | **Number** | **Dilutions** |
| --- | --- | --- | --- |
| ANXA2 | CST | #8235 | 1/1000 |
| MYO1C | abcam | ab194828 | 1/1000 |
| TRIM65 | Novus Biologicals | H00201292-B01P | 1/1000 |
| P65 | abcam | ab32536 | 1/1000 |
| p-P65 | abcam | ab183559 | 1/1000 |
| GAPDH | abcam | ab8245 | 1/1000 |
| β-actin | abcam | ab8226 | 1/1000 |
