# Supplementary material for: METTL3-mediated deficiency of lncRNA HAR1A drives non-small cell lung cancer growth and metastasis by promoting ANXA2 stabilization
Source: Cell Death Discov. 2024 Apr 30;10:203. doi: 10.1038/s41420-024-01965-w (PMC11061277; doi:10.1038/s41420-024-01965-w)
Supplement: Supplementary file 3 — Table S2 [file 41420_2024_1965_MOESM3_ESM.docx]

**Table S2. Mass spectrometry of proteins pulled-down by lncRNA HAR1A in A549 extracts**

| **No.** | **Description** | **Gene names** | **Intensity NC** | **Intensity Probe** | **probe/NC** |
| --- | --- | --- | --- | --- | --- |
| 1 | LIM domain and actin-binding protein 1 OS=Homo sapiens OX=9606 GN=LIMA1 PE=1 SV=1 | LIMA1 | 145660 | 68808000 | 472.3877523 |
| 2 | Cytospin-A OS=Homo sapiens OX=9606 GN=SPECC1L PE=1 SV=2 | SPECC1L | 450180 | 195500000 | 434.2707361 |
| 3 | Myosin-9 OS=Homo sapiens OX=9606 GN=MYH9 PE=1 SV=4 | MYH9 | 258810000 | 96502000000 | 372.8681272 |
| 4 | Annexin A2 OS=Homo sapiens OX=9606 GN=ANXA2 PE=1 SV=2 | ANXA2 | 80030000 | 15702900000 | 196.2126702 |
| 5 | Myosin-14 OS=Homo sapiens OX=9606 GN=MYH14 PE=1 SV=2 | MYH14 | 10980000 | 1956100000 | 178.151184 |
| 6 | Uveal autoantigen with coiled-coil domains and ankyrin repeats OS=Homo sapiens OX=9606 GN=UACA PE=1 SV=2 | UACA | 507360 | 75028000 | 147.8792179 |
| **7** | Gelsolin OS=Homo sapiens OX=9606 GN=GSN PE=1 SV=1 | GSN | 3810300 | 361440000 | 94.85867255 |
| 8 | Actin, alpha skeletal muscle OS=Homo sapiens OX=9606 GN=ACTA1 PE=1 SV=1 | ACTA1 | 8909800 | 697730000 | 78.31039978 |
| 9 | Protein flightless-1 homolog OS=Homo sapiens OX=9606 GN=FLII PE=1 SV=2 | FLII | 2576600 | 173290000 | 67.25529768 |
| 10 | Unconventional myosin-Ic OS=Homo sapiens OX=9606 GN=MYO1C PE=1 SV=4 | MYO1C | 9373600 | 466900000 | 49.81010498 |
