# Supplementary figures and images for: METTL3-mediated deficiency of lncRNA HAR1A drives non-small cell lung cancer growth and metastasis by promoting ANXA2 stabilization
Source: Cell Death Discov. 2024 Apr 30;10:203. doi: 10.1038/s41420-024-01965-w (PMC11061277; doi:10.1038/s41420-024-01965-w)

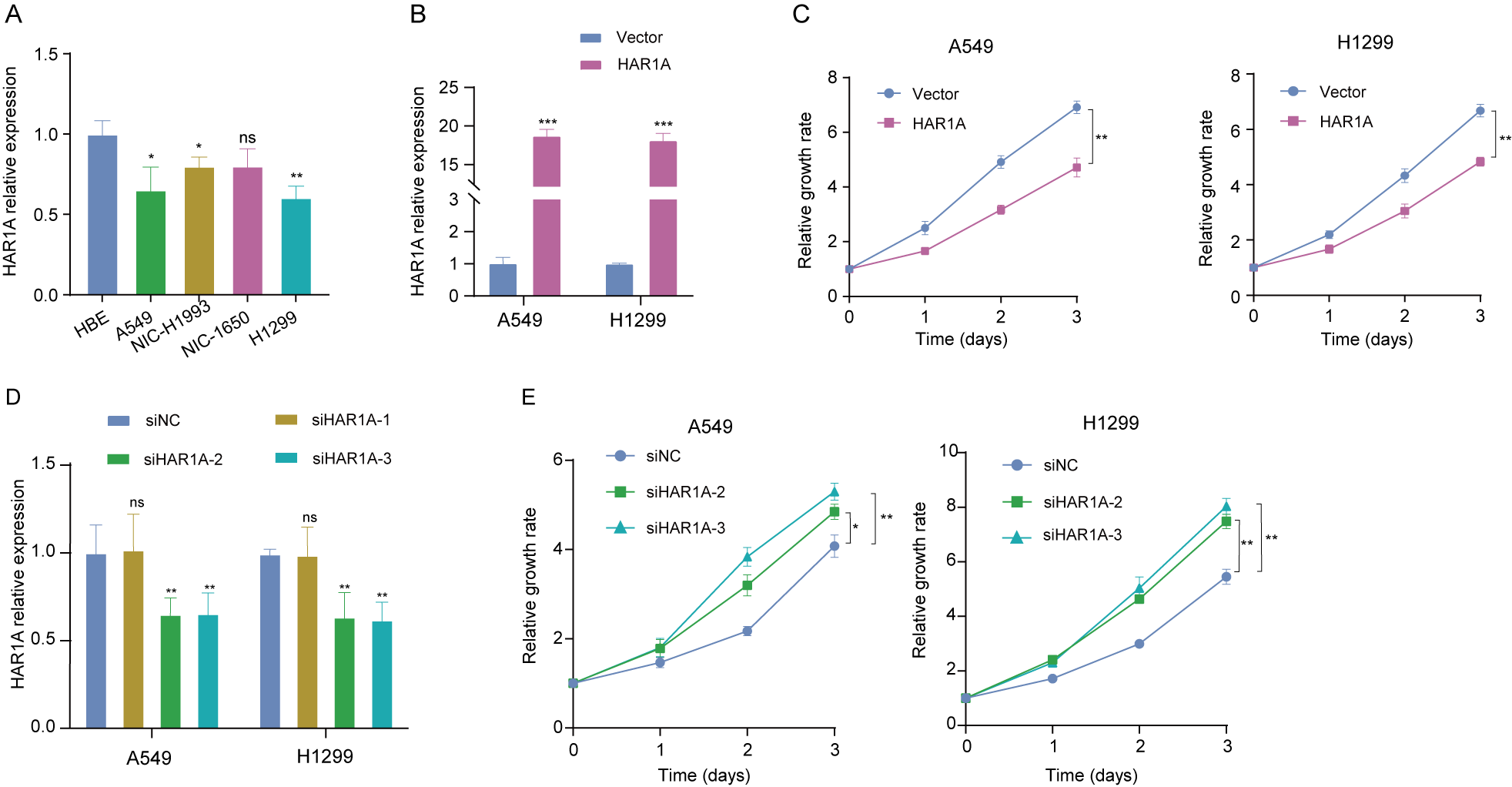

Supplement: Supplementary file 4 — Supplemental Figure 1 [file 41420_2024_1965_MOESM4_ESM.tif]

Fig 5B

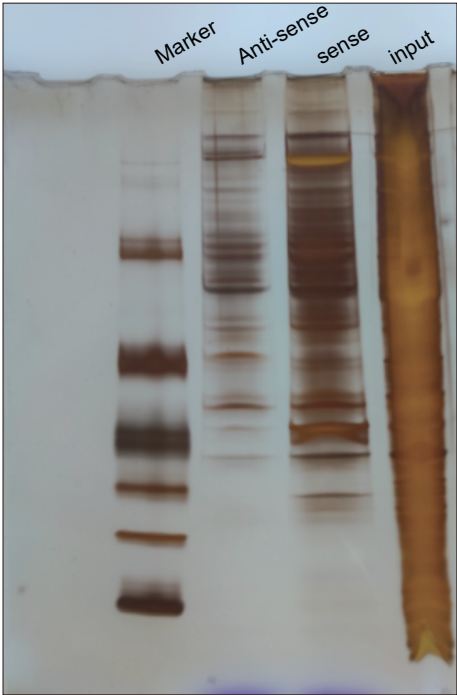

Fig 5D

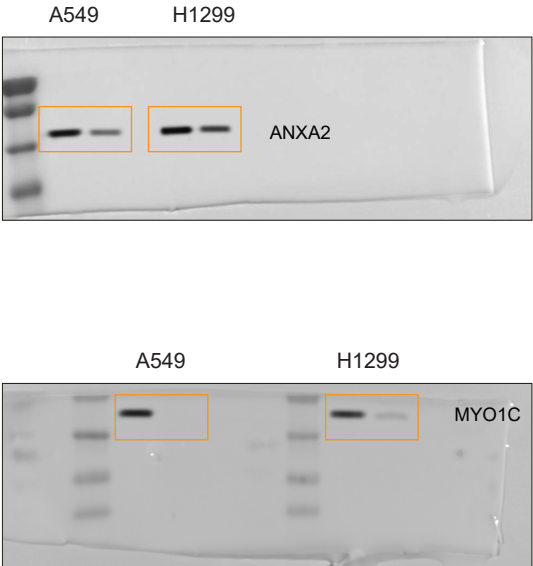

Fig 5E

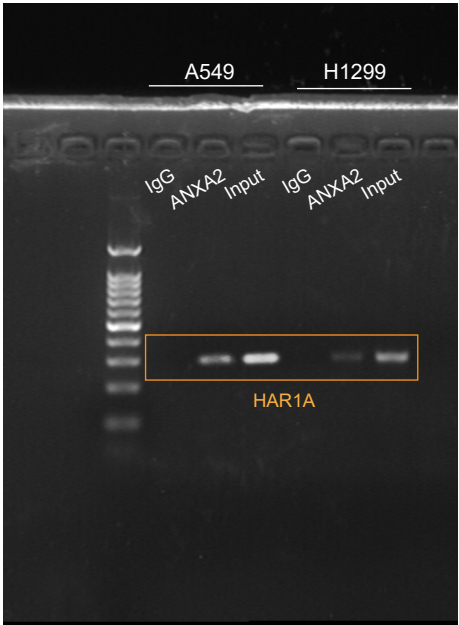

Fig 5H

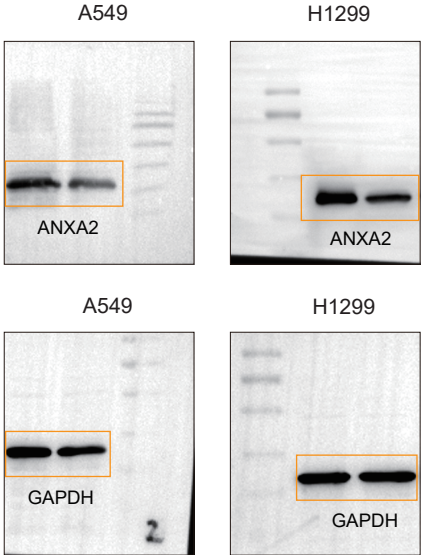

Fig 6A

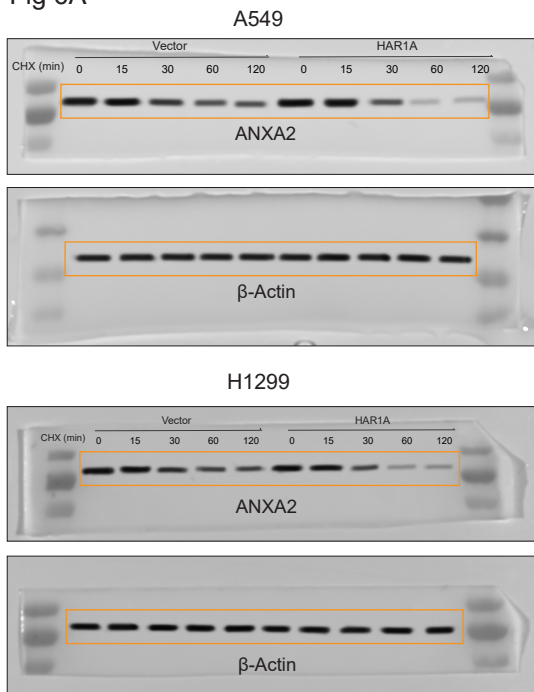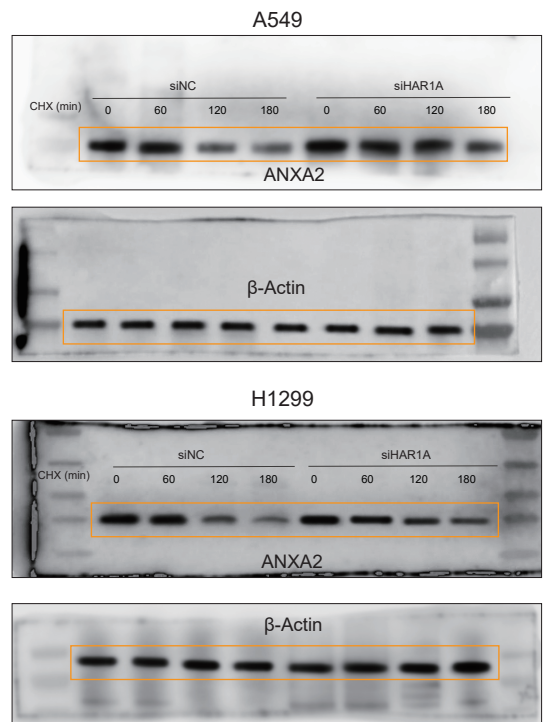

Fig 6B

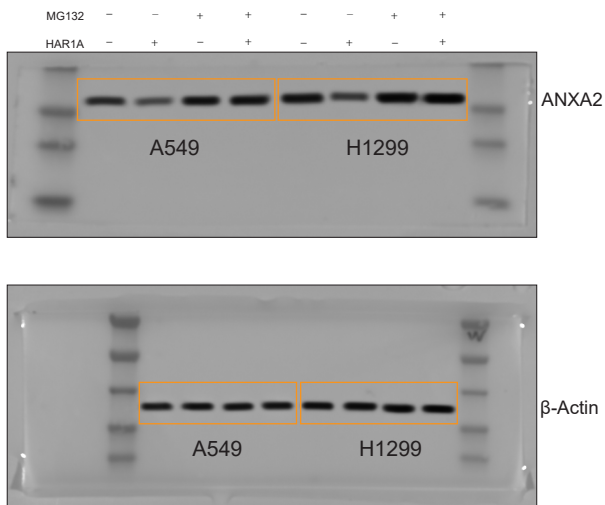

Fig 6C

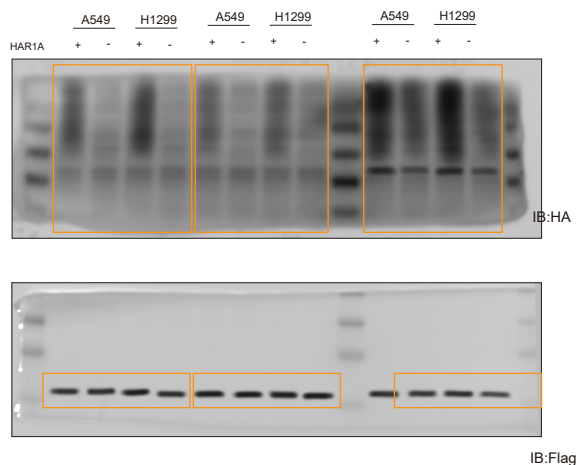

Fig 6E

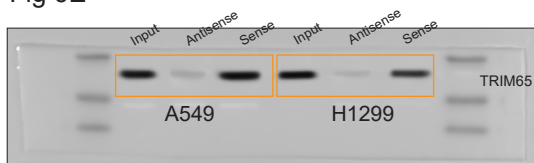

Fig 6G

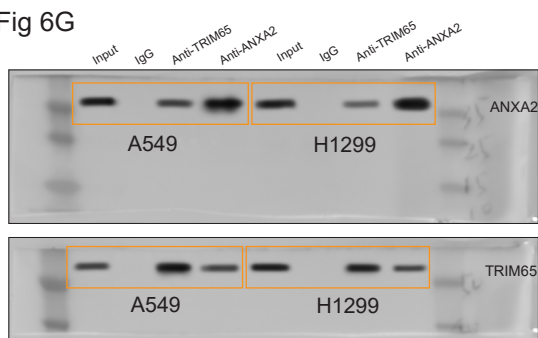

Fig 6H

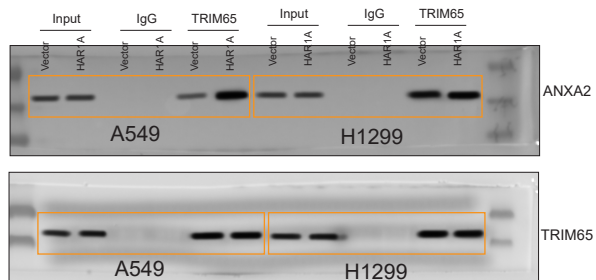

Fig 7D

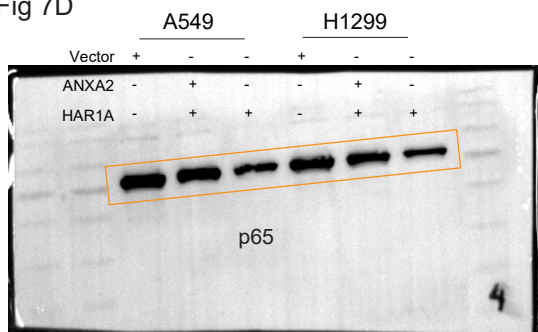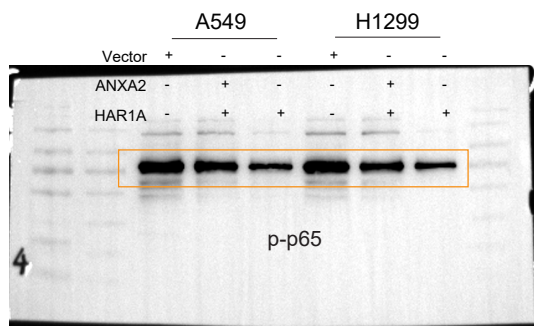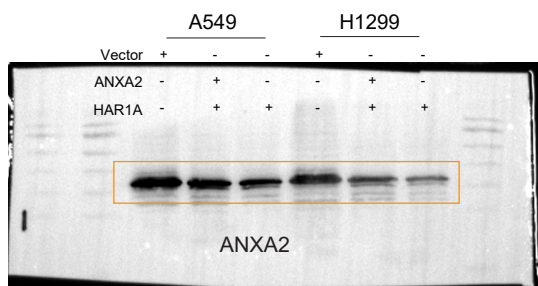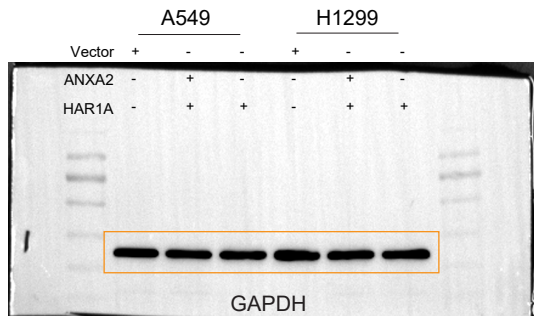

Fig 7H

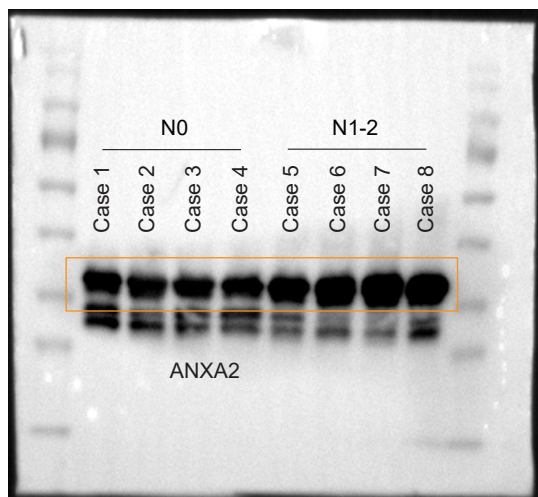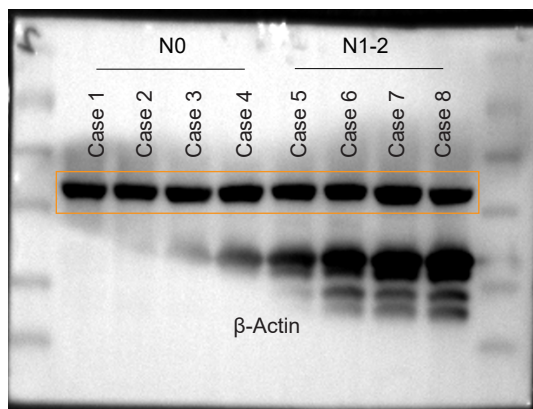

Supplement: Supplementary file 5 — Supplemental Figure 2 [file 41420_2024_1965_MOESM5_ESM.pdf]
