# Supplementary material for: METTL3-mediated deficiency of lncRNA HAR1A drives non-small cell lung cancer growth and metastasis by promoting ANXA2 stabilization
Source: Cell Death Discov. 2024 Apr 30;10:203. doi: 10.1038/s41420-024-01965-w (PMC11061277; doi:10.1038/s41420-024-01965-w)
Supplement: Supplementary file 6 — Figure legends [file 41420_2024_1965_MOESM6_ESM.docx]

**Figure legends**

Figure 1. The influences of *HAR1A* on malignant behaviors of NSCLC cells. **a** Representative images and quantifications indicating scratching assay of *HAR1A*-overexpressing A549 and H1299 stable cells and respective empty vector expressing cells. **b** Immunofluorescent staining was performed to detect the expression of epithelial-mesenchymal transition (EMT) biomarkers, including E-cadherin, N-cadherin, and vimentin. Fluorescence intensity (100%) was measured to represent the expression levels of EMT biomarkers. **c** The mRNA levels of these EMT biomarkers were also evaluated using RT-qRCR. **d** The wound healing assay indicated that gaps almost disappeared in si*HAR1A* cells compared with unfilled gaps in siNC cells at 36h after scratching, suggesting that silencing *HAR1A* promoted NSCLC cell migration. *p < 0.05; **p < 0.01; ***p < 0.001; ****p < 0.0001.

Figure 2. *HAR1A* sensitizes NSCLC cells to paclitaxel (PTX). **a** A549 and H1299 cells were infected lentivirus (LV) empty vectors and LV overexpressing HAR1A (LV-HAR1A) and incubated with increasing concentrations of PTX. Cell viability was checked after 24 h using CCK8 assay. IC50 was calculated in both A549 and h1299 cells. The following experiments were performed using LV-vector and LV-*HAR1A* cells in the presence or absence of PTX. **b** *HAR1A* further aggravated PTX’s colony formation-inhibiting effects on NSCLC cells, suggesting that *HAR1A* might have acted as a chemosensitizer. **c** Edu incorporation assays were used to monitor dividing cells. With the percentage of EdU-labeling cells used as an estimate for proliferation rate, PTX exhibited significantly stronger inhibitory effects on LV-*HAR1A* NSCLC cells than on vector control cells. **d** TUNEL assay revealed that PTX treatment triggered significantly more cells to undergo apoptosis in LV-*HAR1A* NSCLC cells than in vector control cells. *p < 0.05; **p < 0.01; ***p < 0.001; ****p < 0.0001.

Figure 3. *HAR1A* enhances the cytotoxic effects of paclitaxel (PTX) on NSCLC cells and inhibits tumor metastasis in vivo. Impacts of PTX and *HAR1A* overexpression on tumor growth of PTX-treated xenograft mice. **a** The workflow of in vivo tumor growth study. The effect of PTX on the growth of tumors originated from stable cells infected with lentiviruses containing *HAR1A* cDNA (*HAR1A*) or empty lentivirus vectors (vector). Paclitaxel was administered at a dose of 15 mg/kg once every four days. **b** Representative images of mice bearing subcutaneous tumor xenografts for indicated groups and resected tumors from corresponding mice. **c** Tumor growth curves (volume) in mice subcutaneously injected with LV-*HAR1A* or LV-vector stably infected H1299 cells with or without PTX inventions. **d** Tumor weight in mice injected with H1299 stable LV-*HAR1A* or LV-vector cells at day 28 after PTX treatment. **e, f** Edu and TUNEL assays were performed to detect proliferation and apoptosis in metastatic nodes, respectively, followed by quantifications. **g** The workflow of in vivo tumor metastasis study. **h, i** Ex vivo mouse lungs elucidated that reduced lung metastasis in mice injected with *HAR1A*-overexpressing NSCLC cells, compared with mice receiving vector cells. HE and Ki67 stains showed metastatic nodes and proliferating cells. **j, k** Edu and TUNEL assays on metastatic nodes and quantifications. *p < 0.05; **p < 0.01; ***p < 0.001; ****p < 0.0001.

Figure 4. *HAR1A* interacts with the oncogenic protein ANXA2. **a** RT-qPCR was performed to quantify the mRNA levels of *HAR1A,* GAPDH, and U6 in nuclear and cytoplasmic fractions of A549 and H1299 cells. **b** RNA pull-downs were performed using biotinylated antisense and sense sequences of *HAR1A*. Co-precipitated proteins were resolved in SDS-PAGE and visualized with silver staining. **c** Identification of ANAX2 in precipitants of RNA pull-down assay by LC/MS. **d** Following the *HAR1A* RNA pull-down assay, western blot results showed the presence of ANXA2 among the pulled-down proteins. **e** RIP analysis with anti-ANXA2 antibody was conducted in A549 and H1299 cells to show co-precipitated *HAR1A* using RT-qPCR. **f** The representative photograph showing fluorescence in situ hybridization (FISH) with a probe against *HAR1A* and immunofluorescent staining with anti-ANXA2 antibody in A549 and 1299 cells. Red, green, and blue represents biotin-labeled probe against *HAR1A*, immunofluorescent staining of ANXA2, and DAPI staining of the nucleus, respectively. Yellow in the merged image indicates the colocalization of *HAR1A* and ANXA2 in the cells. **g** RT-qPCR analysis of the ANXA2 mRNA levels in LV-vector and LV-*HAR1A* treated A549 and H1299 cells. **h** Immunoblotting to examine ANXA2 levels in A549 and H1299 stable cells overexpressing *HAR1A* or control cells expressing LV-vector. **i** Gene set enrichment analysis (GSEA) to explore the downstream pathway of *HAR1A*. *p < 0.05; **p < 0.01; ***p < 0.001; ****p < 0.0001.

Figure 5. E3 TRIM65 mediates the ubiquitination and degradation of ANXA2. **a** Cells transfected with siNC or si-*HAR1A*, as well as cells with stable *HAR1A* overexpression, were treated with cycloheximide (CHX) for the specified periods of time. Subsequently, cell lysates were analyzed by western blot to examine the half-life of ANXA2 protein. Protein band intensities were quantified using Image J software. **b** Western blot was performed to check the effects of proteasome inhibitor MG132 on the ANXA2 expression levels. **c** HA-Ub and Flag-ANXA2 plasmids were co-introduced into stable A549 and H1299 LV-vector and LV-*HAR1A* cells. Cells were treated with MG132. Immunoprecipitation was performed with cell lysates using an anti-Flag antibody and western blot followed with an anti-Flag or anti-ubiquitin antibody. **d** Predicated E3 ligases for ANAX2 using the UbiBrowser tool. **e** *HAR1A* RNA pull-down assay and western blot showed the interaction between *HAR1A* and TRIM65. **f** RIP analysis coupled with RT-qPCR indicated that TRIM65 could precipitate *HAR1A* in A549 and H1299 cells. **g** Co-immunoprecipitation (Co-IP) of ANXA2 and TRIM65 in A549 and H1299 cells. Western blot with anti-ANXA2 antibody was used to probe the ANXA2 in the anti-TRIM65 antibody-mediated immunoprecipitates. Reverse co-IP was also performed to testify the immunoprecipitation of TRIM65 with anti-ANXA2 antibody. **h** Co-IP revealed that *HAR1A* facilitated the interaction between ANXA2 and TRIM65. *p < 0.05; **p < 0.01; ***p < 0.001; ****p < 0.0001.

Figure 6. *HAR1A* diminished the malignant behaviors of A549 and H1299 cells by downregulating ANXA2. stable A549 and H1299 LV-*HAR1A* cells were further transfected with ANXA2 plamsids. **a** Representative image of crystal violet-stained A549 and H1299 cells with modified expression of *HAR1A* and ANXA2 in the transwell assays. Quantification of migrated and invaded cells indicated that transfection of ANXA2 plasmids partially counteracted *HAR1A*-induced repression of tumor cell migration and invasion. **b** CCK8 was used to measure proliferation of A549 and H1299 cells transduced with LV-vector, LV- *HAR1A+*ANXA2 plasmid, and LV- *HAR1A+*empty plasmid. **c** Gene set enrichment analysis (GSEA) showed the downstream pathway of ANXA2. **d** Western blot to determine the protein levels of p65, p-p65, and ANXA2 in A549 and H1299 cells with indicated gene manipulations. Tumor samples were collected from eight NSCLC patients, along with matched normal tissues. **e** Chest computed tomography imaging of the eight NSCLC patients. The red circles highlight primary lung tumor and the blue arrows indicate regional lymph nodes with pathologically proven metastasis. According to the 8th edition of AJCC TNM staging, N category was defined as N0 (no metastatic lymph node), N1 (metastasis in ipsilateral peribronchial ans/or ipsilateral hilar lymph nodes and intrapulmonary nodes ), N2 (metastasis in ipsilateral mediastinal and/or subcarinal lymph nodes). **f** qRT-PCR was used to measure *HAR1A* in normal tissues versus tumors. **g** Comparsion of *HAR1A* in N0 versus N1-2 tumors. **h** Western blot to compare the ANXA2 levels between N0 and N1-2 tumors. **i** ANXA2 immunohistochemistry staining in NSCLC tumor samples. **j, k** Kaplan-Meier survival curves for lung cancer patients dichotomized by the levels of *HAR1A* or ANXA2, using the K-M plotter. *p < 0.05; **p < 0.01; ***p < 0.001; ****p < 0.0001.

Figure 7. METTL3-mediated m^6^A modifications lead to *HAR1A* degradation. **a, b** RT-qPCR was performed to investigate the effects of overexpression and knockdown of METTL3 on the *HAR1A* levels in A549 and H1299 cells. **c** After inhibiting RNA synthesis with actinomycin D, *HAR1A* degraded faster in NSCLC cells with METTL3 overexpression than in control vector cells at different times. **d** m^6^A RIP coupled with RT-qPCR showed that *HAR1A* was subjected to m^6^A modification, and significantly more m^6^A-modified *HAR1A* RNAs were enriched in A549 and H1299 than in HBE cells. **e** Cells were transduced with siRNAs targeting YTHDF1, YTHDF2, YTHDF3, and scramble controls. siYTHDF2 treatment reduced *HAR1A* levels as shown by RT-qPCR. **f** In cells where RNA synthesis was blocked with actinomycin D, YTHDF2 siRNA slows down the degradation of *HAR1A*. **g** RIP assay, followed by RT-qPCR, revealed the precipitation of YTHDF2 with *HAR1A*. **h** Schematic diagram of molecular mechanisms. *p < 0.05; **p < 0.01; ***p < 0.001; ****p < 0.0001.

Supplemental Figure 1. Effects of *HAR1A* on proliferation of NSCLC cells. **a** The baseline expression of *HAR1A* in normal HEB and labeled NSCLC cell lines. **b** Stable cell lines were generated with lentiviruses expressing *HAR1A* or empty vectors and validated by qRT-PCR. **c** The effects of *HAR1A* on cell proliferation were evaluated using CCK8 assay. **d** *HAR1A* knockdown efficiency was determined by RT-qPCR. **e** CCK8 assay demonstrated that A549 and H1299 cells with *HAR1A* knockdown showed increased proliferation compared to respective siNC-transduced cells. *p < 0.05; **p < 0.01; ***p < 0.001; ****p < 0.0001.
